# Failure to Thrive With Severe Hypokalemia Yields Cystinosis Diagnosis in a 19-Month-Old Female Child: A Case Report

**DOI:** 10.7759/cureus.63122

**Published:** 2024-06-25

**Authors:** Himbert J Sinopoli, Yousef Khouri

**Affiliations:** 1 College of Medicine, Alabama College of Osteopathic Medicine, Dothan, USA; 2 Pediatrics, Alabama College of Osteopathic Medicine, Dothan, USA

**Keywords:** acid-base disorders, cystinosis, fanconi’s anemia, pediatric nephrology, refractory hypokalemia, orphan diseases, pediatrics metabolic diseases

## Abstract

Cystinosis is a rare, genetically inherited disease that affects lysosomal storage of cysteine. It is the most common cause of Fanconi syndrome. Mutations have led to early-onset end-stage renal disease as well as other systemic organ failures. In this case, we report a 19-month-old female child who presented acutely to the outpatient clinic with nausea, vomiting, and diarrhea. The patient was previously diagnosed with unspecified renal tubular acidosis and treated with oral electrolytes. Early labs during her acute presentation showed severe hypokalemia and electrolyte imbalance, which necessitated a transfer to a pediatric ICU. Through confirmatory testing, a diagnosis of cystinosis was made. This case is an example of the recognition and treatment of a rare inherited disease.

## Introduction

Cystinosis is an inherited genetic mutation in the lysosomal transporter carrier protein (CTNS). Mutations in CTNS are the cause of this autosomal recessive transmitted disease. Failure of the carrier protein leads to systemic overload of cysteine [1]. Its incidence is estimated to be between 1:100,000 and 1:200,000 live births and it presents as Fanconi syndrome [2]. Untreated cystinosis affects many organ systems and can lead to end-stage renal disease (ESRD) as early as the end of the first decade of life. There are three different forms of cystinosis, which correspond with both the progression and age of the first symptoms. The infantile form is both the most frequent and most severe. It manifests between 6 and 12 months. The juvenile type is diagnosed later, between 10 and 12 years, and has similar symptoms to the infantile form, albeit with a milder course. The adult form is unique in that it affects the ocular system and spares the renal system. 

Treatment involves both specific and supportive care. The mainstay of treatment is cysteamine. This reduces the accumulated cysteine in lysosomes and the altered molecule leaves affected cells through a different transporter, PQLC2, thus bypassing the mutation. Supportive care is aimed at both hydration and replenishment of electrolytes to avoid more severe consequences [1].

## Case presentation

**Figure 1:**
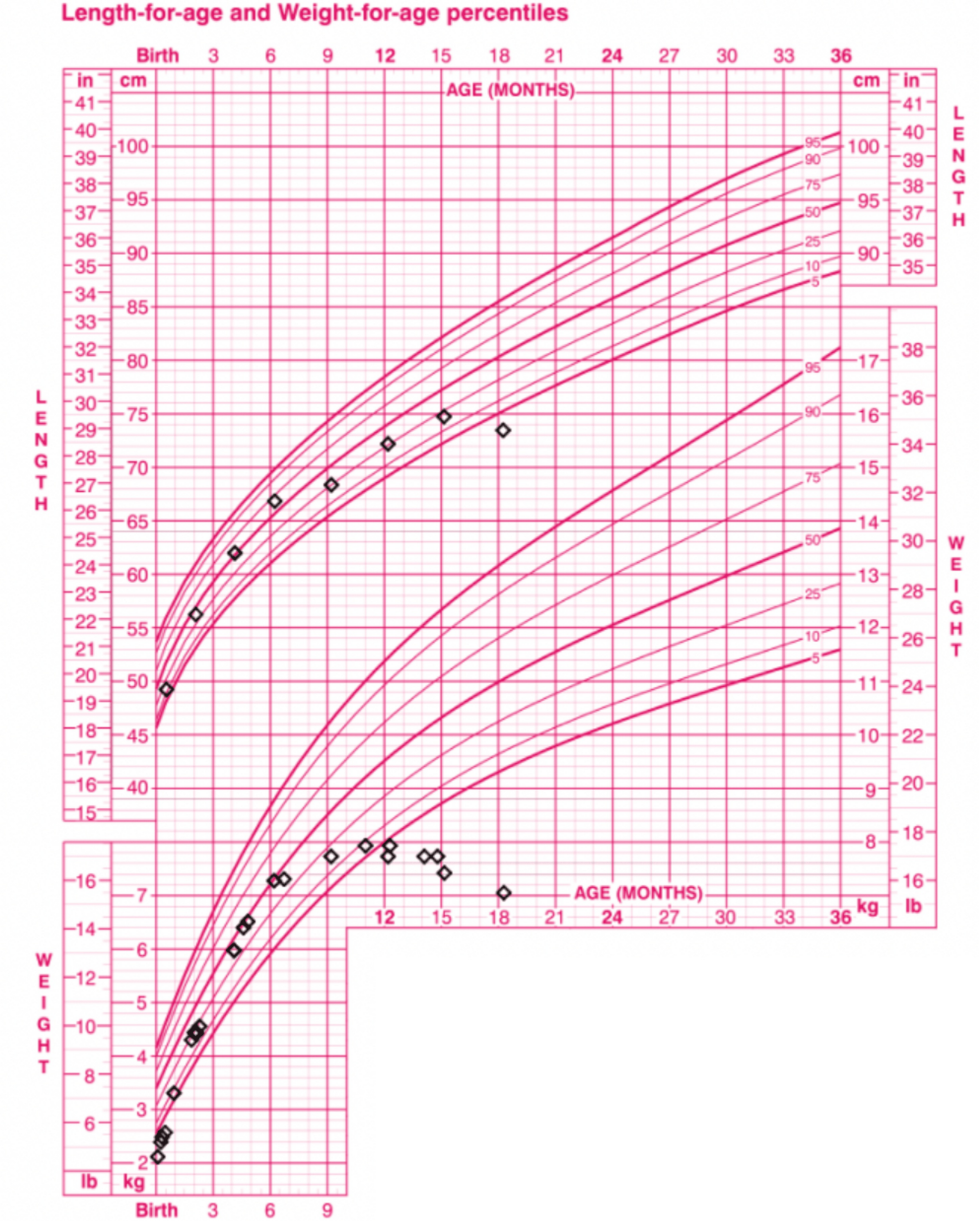
Growth chart of the patient Age in months is plotted on the X-axis. Length, measured from head to toe, is plotted on the top chart on the Y-axis. Likewise, the weight taken at each visit is plotted on the bottom chart on the Y-axis. Corresponding percentiles can be found on the right end of the trend lines

A 19-month-old female child presented to the outpatient clinic for gastroenteritis. Previous medical history was significant for failure to thrive (FTT) and renal tubular acidosis (RTA). FTT was documented with the patient being under the third percentile for height and weight based on age (Figure 1). Discussions centered around increasing caloric density as well as routine lab work. Results from a complete metabolic panel displayed severe metabolic acidosis and entailed a nephrology consultation. RTA was suspected, and she was placed on Oracit. Oracit, a sodium citrate formulation, was prescribed by a consulting pediatric nephrologist. Increasing dosages up to 15 mL three times daily only slightly improved the electrolyte imbalance.

Subjectively, the patient presented with fever and vomiting for two days. The family reported polydipsia and polyuria, with the patient consuming approximately 56 ounces of water daily. She experienced no difficulty breathing or altered mental status. On physical examination, the patient appears frail and fatigued, with fair complexion of hair and eyes. Tracks with eyes and in no acute distress. She does not appear volume-depleted. Slight tachypnea and mild-grade fever were noted. Otherwise, no abnormalities were observed on the head, eyes, ears, nose, and throat (HEENT), cardiopulmonary, musculoskeletal, or abdominal exams. Stat labs were ordered and are shown in Table 1.

**Table 1:** Stat labs drawn from the day of the visit BUN: Blood urea nitrogen; SGOT: Serum glutamic oxaloacetic transaminase; SGPT: Serum glutamic pyruvic transaminase Bolded values indicate lab abnormalities

Test	Result	Reference
Sodium	125	136-145 mmol/L
Potassium	<1.5	3.5-5.1 mmol/L
Chloride	88	98-107 mmol/L
Bicarbonate	22	20-28 mmol/L
Anion gap	15	
Glucose	91	60-110 mg/dL
BUN	9	5-18 mg/dL
Creatinine	0.51	0.2-0.6 mg/dL
BUN/creatinine ratio	18	
Calcium	9.5	9.0-11.0 mg/dL
Total protein	8.1	4.8-7.8 g/dL
Albumin	5.0	3.0-5.0 g/dL
Globulin	3.1	
Alkaline phosphatase	629	50-270 U/L
SGOT	47	10-30 U/L
SGPT	13	10-36 U/L
Phosphorus	1.4	4.0-8.0 mg/dL

Labs indicated electrolyte imbalance with a high anion gap metabolic acidosis. Because of critical labs, the patient was transferred to the pediatric ER and subsequently ICU for further management. Follow-up with nephrology included genetic testing for cystinosis, which revealed a homozygous gene variant of the CTNS gene with deletion of exons 1-10.

The plan going forward for the patient included discussing with family management and the prognosis for this rare disease. Specific therapy aimed at the depletion of intracellular stores of cysteine. Therapy was initiated with PROCYSBI (cysteamine bitartrate) at 50 mg twice daily, titrated up every two to four weeks with a target dose of 250 mg twice daily. Additionally, volume status, as well as other organ systems, are to be monitored due to whole-body encompassing disease progression

## Discussion

FTT is a clinical finding rather than a diagnosis. As such, underlying factors must be sought in order to correct course and stave off potentially life-altering delays. FTT is defined as either a patient measuring below the fifth percentile for height and weight or falling down two major percentiles on a growth chart. In this case, the patient not only measured below the fifth percentile but also dropped three major percentiles. The most common cause of FTT is inadequate caloric intake. Other potential causes include inadequate absorption, increased metabolism, chronic illnesses, malignancies, or genetic factors. There is no single best test to begin the workup of FTT; however, a low-cost broad approach should include a complete blood count, urinalysis, metabolic profile, and thyroid labs. Additional labs should also be ordered based on patient presentation [3].

One of the potential causes of FTT in children, and one that should be suspected with metabolic acidosis or electrolyte abnormalities, is RTA. A wide and nonspecific diagnosis, RTA can affect the nephron throughout its track to filter and reabsorb waste and nutrients. Furthermore, RTA can be subdivided into four distinct types. Type 1, also known as distal, is due to the failure of the collecting ducts to secrete hydrogen ions. It can be caused by drugs (such as amphotericin B or lithium), gene defects, or autoimmune diseases. It is characterized by alkaline urine and acidic blood. Type 2, or proximal, is caused by a defect in the proximal tubule with impaired bicarbonate reabsorption. Isolated genetic mutations in this transportation are rare and mainly caused by Fanconi syndrome. Fanconi is a global proximal tubular dysfunction that encompasses an array of disease processes, cystinosis included. Type 3 RTA is a mixture of both types 1 and 2. Type 4 differs from the first three types in that it results in hyperkalemia. Drugs such as spironolactone and diseases such as lupus or pyelonephritis can result in type 4 RTA. In addition to electrolyte imbalances within the kidneys, acidosis has an effect on other body systems, one of which can be easily measured in FTT is the skeletal system. Acidosis and abnormal calcium and phosphorus cause a halting of osteoblasts and recruitment of osteoclasts, which can lead to stagnant height and rickets in those experiencing FTT [4].

Cystinosis is the most common cause of Fanconi syndrome. In addition to the kidneys, other systems may be involved, including not only the skeletal system as previously mentioned, but also the eyes. Evidence of cysteine crystals may be found as early as one and a half years old, and the use of cysteamine droplets can delay blindness. Current treatment revolves around the use of cysteamine, which can delay ESRD. Without this, ESRD occurs by the end of the first decade of life; with treatment, this is pushed back to the 20th year of life [5,6]. Of note, cystinosis does not present in kidney allografts. Kidney transplantation in cystinosis improves outcomes, although transplant comes with its own challenges [2].

Treatment revolves around cysteamine, which has been the drug of choice for decades as it directly changes cysteine into a molecule that can be removed from the lysosome. However, the drug is not curative and only delays the progression of ESRD. New research has revolved around inflammatory pathways in the disease. Crystals deposited within lysosomes lead to the production of cytokines and other markers that increase inflammation. Non-steroidal anti-inflammatory drugs (NSAIDs) such as indomethacin have been shown to restore better blood flow to the kidneys. Unfortunately, there is minimal data to support the benefits of these. Other disease-modulating therapies include mitochondrial oxygen scavengers such as Mito-TEMPO and galectin-3 inhibitors. Again, these are promising, but there is limited data on their effectiveness in preventing, or at the least, further delaying ESRD [6].

## Conclusions

Cystinosis is a rare inherited lysosomal disease. It is a multisystem disease but primarily affects the kidneys. Extensive workup for our patient, a 19-month-old with severe metabolic acidosis and electrolyte imbalance, led to the confirmatory diagnosis of cystinosis. The mainstay of treatment included cysteamine, which converts cysteine crystals to a form that can be excreted from the cell, delaying the onset of ESRD. Kidney transplants are also indicated when ESRD occurs. Current drug studies focus on reducing inflammation related to crystals in the disease and delaying disease progression.
